# Genetic polymorphisms associated with fatty liver disease and fibrosis in HIV positive patients receiving combined antiretroviral therapy (cART)

**DOI:** 10.1371/journal.pone.0178685

**Published:** 2017-06-08

**Authors:** Leona Dold, Carolin Luda, Carolynne Schwarze-Zander, Christoph Boesecke, Cordula Hansel, Hans-Dieter Nischalke, Philipp Lutz, Raphael Mohr, Jan-Christian Wasmuth, Christian P. Strassburg, Jonel Trebicka, Jürgen Kurt Rockstroh, Ulrich Spengler

**Affiliations:** 1 Department of Internal Medicine I, Rheinische Friedrich-Wilhelms University Bonn, Bonn, Germany; 2 German Center for Infection Research (DZIF), partner site Cologne-Bonn, Bonn, Germany; 3 Department of Internal Medicine III, University of Aachen, Aachen, Germany; Medizinische Fakultat der RWTH Aachen, GERMANY

## Abstract

Hepatic steatosis can occur with any antiretroviral therapy (cART). Although single nucleotide polymorphisms (SNPs) have been identified to predispose to alcoholic and non-alcoholic fatty liver disease, their role for treatment-associated steatosis in HIV-positive patients remains unclear. We determined the frequency of *PNPLA3* (rs738409), *CSPG3/NCAN* (rs2228603), *GCKR* (rs780094), *PPP1R3B* (rs4240624), *TM6SF* (rs8542926), *LYPLAL1* (rs12137855) and *MBOAT7* (rs626283) by RT-PCR in 117 HIV-positive patients on cART and stratified participants based on their “controlled attenuation parameter” (CAP) into probable (CAP: 215–300 dB/m) and definite (CAP >300 dB/m) hepatic steatosis. We analyzed CAP values and routine metabolic parameters according to the allele frequencies. Sixty-five (55.6%) and 13 (11.1%) patients were allocated to probable and definite steatosis. CAP values (p = 0.012) and serum triglycerides (p = 0.043) were increased in carriers of the *GCKR* (rs780094) A allele. Cox logistic regression identified triglycerides (p = 0.006), bilirubin (p = 0.021) and BMI (p = 0.068), but not the genetic parameters as risk factors for the occurrence of hepatic steatosis. Taken together, according to the limited sample size, this exploratory study generates the hypothesis that genetic polymorphisms seem to exert minor effects on the risk for fatty liver disease in HIV-positive patients on cART. Nevertheless, SNPs may modify metabolic complications once metabolic abnormalities have developed. Hence, subsequent analysis of a larger cohort is needed.

## Introduction

Highly active antiretroviral therapy has dramatically reduced death rates from opportunistic diseases in HIV infection but is frequently complicated by dyslipidaemia and fatty liver disease [1]. Although high-risk drugs such as didanosine and stavudine are rarely used nowadays, an increased risk of fatty liver disease is still being observed with antiretroviral therapy. Liver enzyme abnormalities occur in up to 30% of patients infected with the human immunodeficiency virus (HIV), and about 40% of these patients show features of hepatic steatosis with elevated CAP values when examined by controlled attenuation parameter (CAP) [2, 3]. A recent study even reported hepatic steatosis in up to 55% of HIV positive patients, when individuals with elevated liver enzymes were studied by liver biospies [4]. Consequently, chronic liver disease and liver-related mortality remain pivotal challenges in HIV infected patients on antiretroviral therapy [5].

In HIV-negative patients with alcoholic (ASH) and non-alcoholic steatohepatitis (NASH) single-nucleotide polymorphisms in the genes *PNPLA3* (rs738409), *CSPG3/NCAN* (rs2228603), *GCKR* (rs780094), *PPP1R3B* (rs4240624), *LYPLAL1* (rs12137855), *TM6SF2* (rs8542926) and *MBOAT7* (rs626283) have been identified as risk factors for fatty liver disease, liver fibrosis and liver cancer [6–11]. In addition, distinct patterns of serum lipids and glycaemic traits were associated with these polymorphisms [11–14]. Nevertheless, the pathomechanisms underlying these genetic associations are still not fully understood [15].

A recent study on 62 patients with elevated liver aminotransferases reported the *PNPLA3* polymorphism to be a risk factor for hepatic steatosis in HIV-infected individuals [4]. However, our early pilot trial in 57 HIV-infected patients did not support any association between genetic risk factors and hepatic steatosis [16]. Thus, it remains unclear if genetic variants modify the risk for drug-related fatty liver disease or just reflect genetic factors of obesity and metabolic abnormalities in ASH and NASH. To clarify this issue we expanded our pilot study using transient elastography and the controlled attenuation parameter (CAP) as surrogate markers of hepatic fibrosis and steatosis [17].

We report an exploratory study of 117 HIV-positive patients to check if genetic variants predisposing to alcoholic steatosis and NASH also modify liver disease on antiretroviral therapy.

## Patients and methods

### Patients

In 2013, we decided to expand our pilot study on hepatic steatosis and prospectively recruited HIV-infected patients of Caucasian descent, who had been on cART for at least one year before study entry, at the outpatient clinic at the Bonn University Department of Internal Medicine. The patients were monitored every 3 months, and antiretroviral therapy had been advised and adapted following the guidelines for antiretroviral treatment recommended by the European AIDS clinical society (EACS). Before study entry, written consent for all study procedures was obtained from all participants. The study was approved by the local ethics committee of the University of Bonn (decision number 069/10) and was in agreement with the 1975 Declaration of Helsinki. The study was supported by a grant from the German center for infection research (DZIF).

All patients underwent careful clinical examination and measurement of CAP and liver stiffness by transient elastography. Blood counts and liver-function tests (alanine and aspartate aminotransferases (ALT, AST), alkaline phosphatase (AP), bilirubin, and y-glutamyltranspeptidase (GGT), as well as parameters of fat and glucose metabolism such as total cholesterol, high density lipoprotein (HDL), low density lipoprotein (LDL), cholesterol, triglycerides and HbA1c were determined by routine biochemical procedures. Patients with a replicative hepatitis C virus infection or positive HBs Ag were excluded from the study. Alcohol abuse was ruled out by questionnaire and patients with an alcohol consumption above 30 g/week for men and 20 g/week for women were excluded from the study.

One hundred-forty-nine healthy blood donors, anonymized for ethical reasons, served as control group. Only information about age, gender and negative HBV, HCV and HIV status information was available from healthy blood donors. Further 133 individuals with proven alcoholic liver cirrhosis served together with the healthy controls as a reference for the allele distribution in the background population and as disease controls to check validity of our analysis, respectively. All subjects were of Caucasian descent.

### Diagnosis of HIV infection and viral hepatitis

HIV load was determined quantitatively using the Abbott RealTime m2000rt (Abbott Laboratories, Illinois, USA). This assay has a detection limit of 40 copies/mL. Additionally, chronic viral hepatitis was assessed by routine assays for hepatitis B surface antigen, HBV-DNA, HCV-RNA and HCV antibodies.

### Determination of SNP alleles

Genomic DNA was extracted from 200 μl EDTA-blood using the QIAamp Blood Mini Kit (Qiagen, Hilden, Germany) according to the manufacturer’s protocol. Determination of the *PNPLA3* (rs738409), *CSPG3/NCAN* (rs2228603), *GCKR* (rs780094), *PPP1R3B* (rs4240624), *TM6SF* (rs8542926), *LYPLAL1* (rs12137855) and *MBOAT7* (rs626283) polymorphisms was performed by LightCycler real time PCR (Roche, Mannheim, Germany) using commercial LightSNiP (SimpleProbe) assays from TIB-MolBiol (Berlin, Germany) according to the manufacturer’s recommendations. All PCRs for SNPs determination were repeated by two independent investigators (C.L and H.D.N), in order to ensure correct typing results.

### Transient elastography and CAP

Liver stiffness and CAP measurements were performed on a “Fibroscan 502 Touch” device equipped with an M probe (Echosens, Paris, France). We assessed hepatic steatosis via the novel controlled attenuation parameter (CAP) technology [17, 18]. CAP measures the longitudinal attenuation of elastic waves in liver tissue at the center frequency of the FibroScan probe. This non-invasive technique yields results in dB/m, which correspond to intrahepatic fat contents. Liver stiffness results are expressed in kPa. Liver stiffness and CAP were obtained simultaneously in the same volume of liver parenchyma and represent the median of 10 measurements. All CAP measurements were performed by two well trained examiners.

Divergent from our initial pilot study in 2013 we adapted our cut-off values to identify patients with fatty liver disease by CAP according to the refined criteria published in the meantime by Karlas et al. [19]. Thus, patients were classified as having no steatosis when CAP values were <215 dB/m and as having probable hepatic and definite steatosis when CAP values were between 215 and 300 dB/m and above 300 dB/m, respectively.

Liver stiffness was allocated to histological Metavir fibrosis scores with the following boundaries: Stiffness > 7.1 kPa (Metavir F ≥ 2), Stiffness 7.1–12.4 kPa (significant fibrosis). Liver stiffness of ≥12.5 kPa (Metavir F≥ 3–4) was considered as severe fibrosis [20, 21].

### Statistical methods

Genotype frequencies were determined and tested for consistency using a web-based program (http://ihg.gsf.de/cgi-bin/hw/hwa1.pl.). This software calculates De Finetti statistics. In addition, statistical analysis are controlled via several procedures comprising an Exact test and Pearson´s goodness-of fit chi-square and a check for deviation from the Hardy Weinberg Equilibrium in small samples [22]. Allele and genotype frequencies were compared between patient groups and controls by Pearson's goodness-of-fit chi^2^ test and Armitage's trend test, respectively.

In order to identify potential confounding parameters, differences in demographic, viral and biochemical parameters were compared between the patient groups by T-tests, chi-2 or the non-parametric Mann Whitey test as appropriate. Multiple testing was corrected by ANOVA with Bonferoni correction. Cox regression was calculated to identify independent risk factors for steatosis and fibrosis. Parameters with p< 0.1 at univariate analysis (use of PIs, BMI, ALT, AST, yGT, Bilirubin, HbA1c) and the SNPs, respectively, were entered into a forward conditional proportional-hazard regression model. Criteria for inclusion and exclusion of variables in the forward conditional model were p<0.05 and p>0.01 respectively. All calculations were performed with the SPSS 22.0 software package (SPSS, Munich, Germany).

## Results

### Clinical and biochemical characteristics of the patients

We recruited 142 Caucasian HIV-infected patients. Inadvertently 18 and 7 patients had markers of chronic HCV and HBV infection, respectively. Therefore these patients were excluded, leaving 117 patients for the final analysis. Clinical details of the HIV patients as well as available data from the healthy and disease control group (patients with alcoholic cirrhosis) are summarized in Table 1. Risk factors for HIV infection were homosexual and heterosexual contacts in 68 (58.1%) and 39 (33.3%) patients, respectively. Further risk factors for HIV transmission were prior exposure to blood and blood products in two patients (1.7%) and intravenous drug use (IVDU) in six patients (5.1%). In two patients (1.7%) risk factors for HIV transmission could not be identified with certainty.

**Table 1:** Patient characteristics.

	Healthy controls	HIV (all patients)	HIV patients with fatty liver CAP >300 dB/m	HIV-pos. patients with probable fatty liver CAP 215–300 dB/m	HIV-pos. patients without fatty liver CAP < 215 dB/m	HIV-neg. Disease controls (alcoholic cirrhosis)
**Number of patients n (%)**	149 (100)	117 (100)	13 (11.1)	65 (55.6)	39 (33.3)	133 (100)
Age (years)	39.4 (20.0–73)	47.2 (23.9–71.9)	49.3 (30.4–65.5)	47.6 (24.8–69.3)	46.7 (23.9–71.9)	57.9 (29–81.0)
Gender: male/female	90/59	97/20	12/1	55/10	30/9	89/44
**BMI**	N.D.	23.3 (17.3–60.6)	27.5 (18.4–35.5) **A)**	23.3 (17.3–60.6)	21.6 (18.5–33.5)	25.9 (13.8–62.9)
**Number of Patients with detectable HIV (%) and their median viral loads in copies/mL (range)**	0 (N/A)	6 (5.1)	1 (7.6)	5 (7.6)	0 (N/A)	0 (N/A)
	0 (N/A)	81.0 (44–3158)	76 (N/A)	257 (44–3158)	0 (N/A)	0 (N/A)
**AST** [U/L]	N.D.	23.0 (9–93.0)	24.0 (13–59)	23.0 (13–65)	24.0 (9–93)	114.8 (9–3349)
**ALT** [U/L]	N.D.	29.0 (10–99.0)	31.0 (20–84)	29.0 (10–75)	31.0 (13–99)	54.9 (7–2017)
**GGT** [U/L]	N.D.	45.0 (17–302)	64.0 (32–131) **B)**	45.0 (22–302)	41.0 (17–148)	207.9 (24–1277)
**Total bilirubin** [mg/dl]	N.D.	0.45 (0.15–4.58)	0.65 (0.28–4.60)	0.39 (0.15–3.05)	0.46 (0.17–1.46)	3.8 (0.24–38.7)
**Absolut CD4 counts**	N.D.	515.0 (98–1633)	689.0 (311–890)	497.5 (98–1633)	505.5 (271–949)	N.D.
**HDL-cholesterol** [mg/dl]	N.D.	43.0 (18–127)	41.0 (16–58) **C)**	40.0 (22–83)	49.0 (24–127)	N.D.
**LDL-cholesterol** [mg/dl]	N.D.	122.5 (34–206)	142.0 (77–186) **D)**	125.0 (34–206)	115.0 (58–190)	N.D.
**Triglycerides** [mg/dl]	N.D.	161.5 (39–1199)	233.0 (134–1199) **E)**	172.0 (60–672)	108.0 (39–466)	N.D.
**Total Cholesterol** [mg/dl]	N.D.	203.0 (119–278)	234.0 (121–278) **F)**	201.0 (127–276)	197.0 (119–277)	N.D.
**HAART regimens**						
NRTI	N.D.	115 (98.3)	12 (92.3)	38 (58.5)	39 (100)	N.D.
NNRTI	N.D.	72 (61.5)	7 (53.8)	64 (98.5)	27 (69.2)	N.D.
PI	N.D.	74 (63.2)	9 (69.2)	43 (66.2)	22 (56.4)	N.D.
Integrase inhibitors	N.D.	18 (15.4)	3 (23.1)	13 (20.0)	2 (5.1)	N.D.
Entry inhibitors	N.D.	5 (4.3)	1 (7.7)	1 (1.5)	3 (7.7)	N.D.
Prior D-drug exposure	N.D.	22 (18.8)	3 (23.1)	13 (20.0)	6 (15.4)	N.D.
**HAART duration** (months)	N.D.	72.0 (12–216)	96.0 (12–204)	72.0 (12–216)	72.0 (12–204)	N.D.

Data are shown as median (range) and n (%); N.D. = not done; N/A = not applicable

A) *p* = 0.002, B) *p* = 0.049, C) p = 0.043, D) *p* = 0.056, E) *p =* 0.012 F) *p* = 0.054 versus patients without fatty liver.

Median cART duration was 72 months (range 12–216 months). Seventy-two (61.5%) and 74 (63.2%) patients had previous exposure to non-nucleoside reverse transcriptase inhibitors (NNRTI) and protease inhibitor (PI)-based antiretroviral regimes, respectively. Twenty-two (18.8%) individuals had previously taken D-drugs, such as didanosine (DDI) and stavudine (D4T).

Based on their CAP results 65 HIV-positive patients (55.5%) were allocated to have probable fatty liver disease (CAP: 215–300 dB/m), and 13 patients (11.1%) were considered to have definite hepatic steatosis (CAP >300 dB/m). In 39 patients (33.3%) fatty liver disease was excluded (CAP< 215 dB/m). Sixteen (13.7%) patients had relevant hepatic fibrosis F2 (liver stiffness: 7.1–12.4 kPa) and one patient (0.9%%) had severe fibrosis (stiffness: ≥12.5 kPa). Concerning distinct substance classes of antiretroviral therapy, patients with current or prior use of protease inhibitors showed higher serum bilirubin levels (0.786 versus 0.355; p<0.001) than patients without PI exposure. Increased bilirubin, however, could not statistically be attributed to the use of atazanavir in our cohort. Otherwise distribution of antiretroviral regimens did not differ significantly between HIV positive patient groups with different degrees of hepatic steatosis and/or fibrosis. In particular, there were no differences concerning prior exposure to D-drugs, nor duration of cART between the different groups (Table 1).

HIV positive patients with definite hepatic steatosis (CAP >300 dB/m) had significantly higher body-mass-index (BMI) (p = 0.002), GGT (p = 0.049), LDL (p = 0.056), HDL (p = 0.043), cholesterol (p = 0.054) and serum triglyceride levels (p = 0.012) than HIV patients with no or probable steatosis.

### Analysis of gene effects

Taking into account the limited number of individuals in our study, we decided to analyse gene effects only in a dominant model. This approach had enough statistical power as to correctly identify and confirm the known dominant effect of the *PNPLA3* rs738409 variant in our disease control group of patients with alcoholic cirrhosis (Table 2).

**Table 2:** SNPs allele distribution in HIV positive patients, HIV positive patients with fatty liver, healthy controls and alcoholic fatty liver.

	Healthy controls (n = 149)	HIV patients (all) (n = 117)	HIV patients with fatty liver (n = 13)	HIV patients with probable fatty liver (n = 65)	HIV patients without fatty liver (n = 39)	Patients with alcoholic fatty liver (n = 133)
PNPLA3 (rs738409)						
CC	89 (59.7%)	75 (64.1%)	10 (76.9%)	38 (58.5%)	27 (69.2%)	52 (39.1%)
GC	53 (35.6%)	37 (31.6%)	2 (15.4%)	25 (38.5%)	10 (25.6%)	64 (48.1%)
GG	7 (4.7%)	5 (4.3%)	1 (7.7%)	2 (3.1%)	2 (5.1%)	17 (12.8%) A)
CSPG3/NCAN (rs2228603)						
CC	124 (83.2%)	104 (88.9%)	11 (84.6%)	57 (87.7%)	36 (92.3%)	107 (80.4%)
CT	23 (15.4%)	12 (10.3%)	2 (15.4%)	8 (12.3%)	2 (5.1%)	23 (17.3%)
TT	2 (1.3%)	1 (0.9%)	0 (0%)	0 (0%)	1 (2.6%)	3 (2.3%)
GCKR (rs780094)						
GG	53 (35.6%)	41 (35.0%)	3 (23.1%)	18 (27.7%)	20 (51.3%)	49 (36.8%)
GA	77 (51.7%)	60 (51.3%)	10 (76.9%)	34 (52.3%)	16 (41.0%)	64 (48.1%)
AA	19 (12.8%)	16 (13.7%)	0 (0%)	13 (20.0%)	3 (7.7%)	20 (15.0%)
PPP1R3B (rs4240624)						
AA	124 (83.2%)	96 (82.1%)	12 (92.3%)	50 (76.9%)	34 (87.2%)	112 (84.2%)
AG	25 (16.8%)	21 (17.9%)	1 (7.7%)	15 (23.1%)	5 (12.8%)	20 (15.0%)
GG	0 (0%)	0 (0%)	0 (0%)	0 (0%)	0 (0%)	1 (0.8%)
LYPLAL (rs12137855)						
CC	107 (71.8%)	72 (61.5%)	8 (61.5%)	39 (60.0%)	25 (64.1%)	90 (67.7%)
CT	38 (25.5%)	40 (34.2%)	5 (38.5%)	22 (33.8%)	13 (33.3%)	40 (30.1%)
TT	4 (2.7%)	5 (4.3%)	0 (0%)	4 (6.2%)	1 (2.6%)	3 (2.3%)
TM6FS2 (rs8542926)						
CC	125 (83.9%)	103 (88.0%)	11 (84.6%)	56 (86.2%)	36 (92.3%)	105 (78.9%)
CT	22 (14.8%)	13 (11.1%)	2 (15.4%)	9 (13.8%)	2 (5.1%)	23 (17.3%)
TT	2 (1.3%)	1 (0.9%)	0 (0%)	0 (0%)	1 (2.6%)	5 (3.8%)
MBOAT (rs626283)						
CC	nd	38 (32.5%)	3 (23.1%)	19 (29.2%)	16 (41.0%)	Nd
CT	nd	57 (48.7%)	6 (46.2%)	35 (52.3%)	17 (43.6%)	Nd
TT	nd	22 (18.8%)	4 (30.8%)	12 (18.5%)	6 (15.4%)	Nd

A) Patients with alcoholic fatty liver vs. healthy controls p = 0.00018

Frequencies of the minor alleles varied between 8.4% and 38.6% in the background population (S1 Table).

Distributions of all alleles (*PNPLA3* (rs738409), *CSPG3/NCAN* (rs2228603), *PPP1R3B* (rs4240624), *TM6FS2* (rs8542926) and *LYPLAL1* (rs12137855) and *GCKR* (rs780094)) were in the Hardy-Weinberg equilibrium.

Allele frequencies were not significantly different between HIV positive patients with and without fatty liver disease (Table 2).

Next, we compared the distribution of genetic variants between HIV patients with and without hepatic fibrosis (stiffness ≥ 7.1 kPa) to those with normal results (Table 3). Of note, none of the analyzed polymorphisms correlated with the presence of hepatic fibrosis among our HIV positive patients on antiretroviral treatment.

**Table 3:** SNPs allele distribution in HIV positive patients, HIV positive patients with fibrosis, healthy controls and alcoholic fatty liver.

	Healthy controls (n = 149)	HIV patients (all) (n = 117)	HIV patients with relevant fibrosis ≥7.1 kPa (n = 16)	HIV patients without relevant fibrosis <7.1 kPa (n = 101)	Patients with alcoholic fatty liver (n = 133)
PNPLA3 (rs738409)					
CC	89 (59.7%)	75 (64.1%)	11 (68.8%)	64 (63.4%)	52 (39.1%)
GC	53 (35.6%)	37 (31.6%)	4 (25.0%)	33 (32.7%)	64 (48.1%)
GG	7 (4.7%)	5 (4.3%)	1 (6.2%)	4 (4.0%)	17 (12.8%)
CSPG3/NCAN (rs2228603)					
CC	124 (83.2%)	104 (88.9%)	12 (75.0%)	92 (91.1%)	107 (80.4%)
CT	23 (15.4%)	12 (10.3%)	3 (18.8%)	9 (8.9%)	23 (17.3%)
TT	2 (1.3%)	1 (0.9%)	1 (6.3%)	0 (0%)	3 (2.3%)
GCKR (rs780094)					
GG	53 (35.6%)	41 (35.0%)	7 (43.8%)	34 (33.7%)	49 (36.8%)
GA	77 (51.7%)	60 (51.3%)	7 (43.8%)	53 (52.5%)	64 (48.1%)
AA	19 (12.8%)	16 (13.7%)	2 (12.5%)	14 (13.9%)	20 (15.0%)
PPP1R3B (rs4240624)					
AA	124 (83.2%)	96 (82.1%)	12 (75.0%)	84 (83.2%)	112 (84.2%)
AG	25 (16.8%)	21 (17.9%)	4 (25.0%)	17 (16.8%)	20 (15.0%)
GG	0 (0%)	0 (0%)	0 (0%)	0 (0%)	1 (0.8%)
LYPLAL (rs12137855)					
CC	107 (71.8%)	72 (61.5%)	10 (62.5%)	62 (61.4%)	90 (67.7%)
CT	38 (25.5%)	40 (34.2%)	6 (37.5%)	34 (33.7%)	40 (30.1%)
TT	4 (2.7%)	5 (4.3%)	0 (0%)	5 (5.0%)	3 (2.3%)
TM6FS2 (rs8542926)					
CC	125 (83.9%)	103 (88.0%)	12 (75.0%)	91 (90.1%)	105 (78.9%)
CT	22 (14.8%)	13 (11.1%)	3 (18.8%)	10 (9.9%)	23 (17.3%)
TT	2 (1.3%)	1 (0.9%)	1 (6.3%)	0 (0%)	5 (3.8%)
MBOAT (rs626283)					
CC	n.d.	38 (32.5%)	7 (43.8%)	31 (30.7%)	n.d.
CT	n.d.	57 (48.7%)	8 (50.0%)	49 (48.5%)	n.d.
TT	n.d.	22 (18.8%)	1 (6.3%)	21 (20.8%)	n.d.

**Fig 1:**
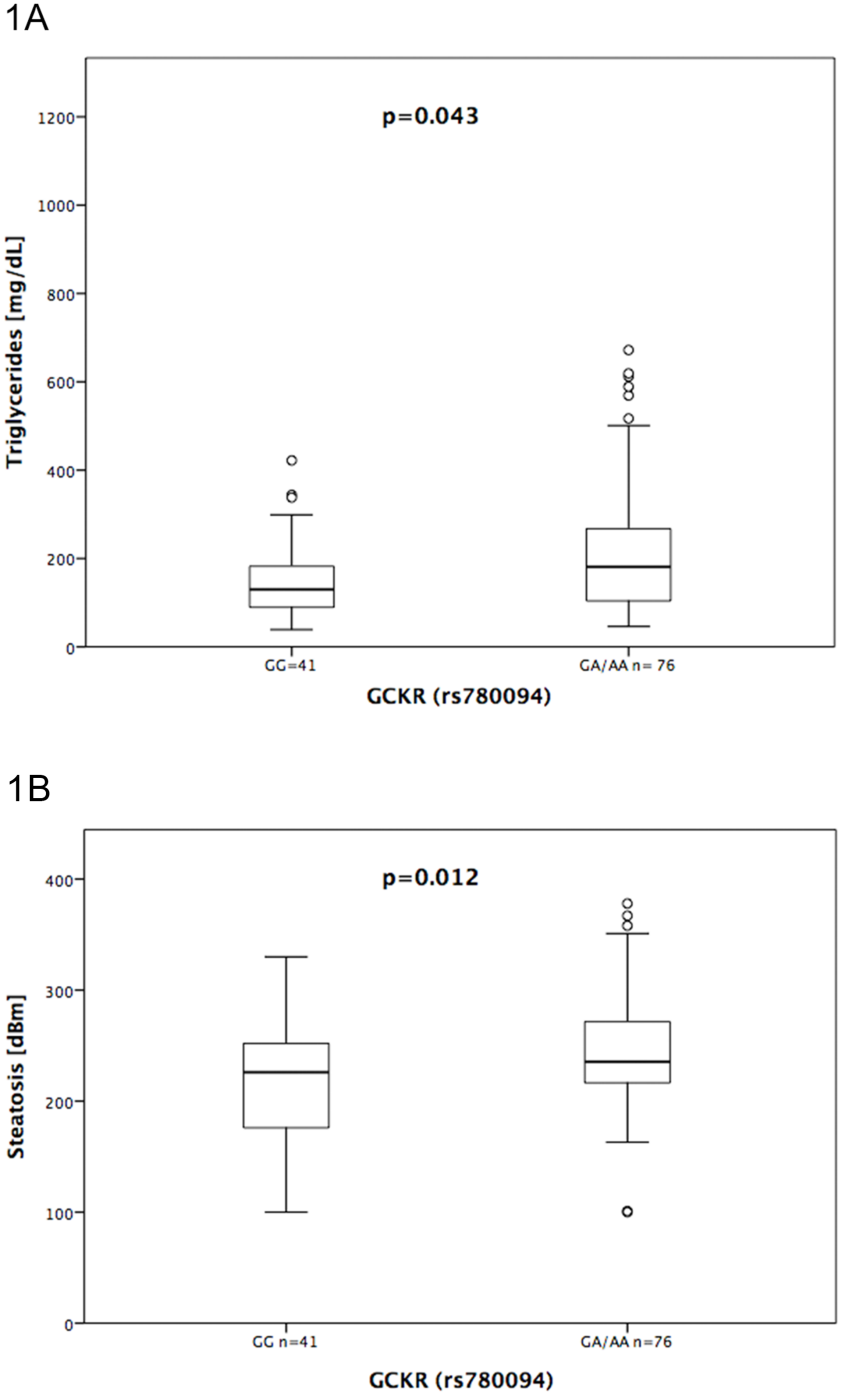
Effects of the GCKR (rs780094) A mutation on hepatic steatosis A) and serum triglyceride levels B) in HIV-positive patients. **A**: Liver fat assessed by the CAP parameter [dB/m] in GCKR (rs780094) wild type homozygous patients versus carriers of the GCKR (rs780094) A mutation. **B**: Levels of serum triglycerides [mg/dl] in wild type homozygous patients versus carriers of the GCKR (rs780094) A mutation. Box and whisker plots indicating medians, 10-, 25-, 75, and 90-percentiles as well as individual outliers.

In addition, we analysed if any of the polymorphisms had disease-modifying effects and studied average levels of steatosis, fibrosis, liver enzymes, bodyweight, BMI and parameters of lipid metabolism. This analysis revealed significantly increased CAP values (p = 0.012) and triglyceride levels (p = 0.043) in carriers of the *GCKR* rs780094 A allele (Fig 1).

Finally, we studied logistic regression models for steatosis (CAP ≥300 db/m) and fibrosis (hepatic stiffness ≥7.1 kPa) using forward conditional analysis on putative determinants at p<0.1 in univariate analysis (use of PIs, BMI, ALT, AST, GGT, bilirubin, HBA1c) and the SNPs, respectively. None of the seven SNPs remained in the final models, while increased BMI (hazard ratio [HR] = 1.103) as well as elevated bilirubin (HR = 2.568) and serum triglyceride (HR = 1.006) remained independent risk factors associated with steatosis. Elevated levels of aspartate aminotransferase (HR = 1.087) were independently associated with significant fibrosis (Table 4).

**Table 4:** Parameters entering the final regression models.

**A) Steatosis (CAP ≥300 dB/m)**:			
Parameter	Level of significance	Hazard ratio	95% Confidence Interval
Triglycerides (mg/dl)	p = 0.006	1.006	(1.002–1.009)
Bilirubin (mg/dl)	p = 0.021	2.568	(1.155–5.710)
BMI (Kg/m^2^)	p = 0.068	1.103	(0.993–1.227)
**B) Fibrosis (Stiffness ≥7.1 kPa)**			
Parameter	Level of significance	Hazard ratio	95% Confidence Interval
AST (IU/ml)	p = 0.006	1.087	(1.027–1.151)

## Discussion

Non-alcoholic fatty liver disease (NAFLD) encompasses a broad spectrum of disorders that includes simple hepatic steatosis, steatohepatitis, fibrosis, cirrhosis and liver cancer [15]. NAFLD is considered a complex disease trait occurring when environmental factors act on a susceptible polygenic background of multiple factors [23]. Recent molecular studies identified several polymorphic genetic loci that seem to determine polygenic susceptibility traits. Such genetic susceptibility variants comprise single nucleotide polymorphisms in *PNPLA3* (rs738409), *CSPG3/NCAN* (rs2228603), *PPP1R3B* (rs4240624), *TM6FS2* (rs8542926), *GCKR* (rs780094) and *LYPLAL1* (rs12137855) [11, 15, 24]. Of note, polymorphisms in *PNPLA3* and *TM6SF* might be “master regulators” of metabolic syndrome outcomes, which potentially determine the full range of NAFLD manifestations from hepatic fat accumulation to liver cancer [15]. Interestingly, the same set of genes and a further polymorphism in the *MBOAT* gene (rs626283) also determine the risk of fatty liver disease and its complications in patients with high alcohol consumption [10], suggesting that a shared mechanism leads to fatty liver damage in metabolic liver disease independently from the underlying causes (NASH versus ASH).

Hepatic steatosis in HIV infection is an even more complex problem, since accumulation of lipids in the liver is the combined result of hepatic drug toxicity, metabolic factors and accelerated aging. It constitutes a prominent problem in HIV-infected patients on effective antiretroviral therapy (cART) [25]. Therefore, we decided to undertake an exploratory study to check if genetic variants associated with liver disease in NAFLD and alcohol consumption also determine hepatic fat content and fibrosis in HIV-infected patients on cART.

Overall, our study on 117 HIV-positive patients on cART does not indicate that genetic polymorphisms predisposing to NAFLD in HIV-negative individuals are also major risk factors for drug-related liver steatosis induced by cART, because we did not detect genetic effects corresponding in strength to the role of the “master regulators” *PNPLA3* and *TM6SF2* in alcoholic and non-alcoholic fatty liver disease. Thus, we decided to stop recruitment, since the available data indicated that potential effects of the seven studied SNPs must be comparatively small. Instead, we found conventional metabolic features such as high BMI, elevated serum triglycerides and cholesterol to be associated with the presence of hepatic steatosis in HIV-positive patients on cART. These findings suggest that the known genetic variants may affect the risk attributable to co-factors such as obesity or alcohol consumption rather than that of drug-induced toxicity itself. This interpretation also reconciles our data with two recent studies reporting associations between the *PNPLA3* polymorphism and steatohepatitis or fibrosis in HIV-positive patients with elevated aminotransferases and in HIV/HCV co-infection respectively. The 62 HIV-positive patients with elevated aminotransferases studied by Morse et al. had an elevated median BMI of 27.6 kg/cm^2^, which was considerably higher than the average BMI in our patients (24.2 kg/cm^2^), which was within the normal range [4]. Both increased BMI and elevated aminotransferases indicate that in this cohort concomitant metabolic factors such as obesity have been more prominent than in our cohort. Jimenez-Sousa and colleges had proposed that the *PNPLA 3* polymorphism is a risk factor for fibrosis development in HIV/HCV co-infected patients [26], while analogous to our study this polymorphism did apparently not predispose to hepatic steatosis. The association between *PNPLA3* variants and fibrosis is likely to reflect effects of HCV co-infection rather than those from drug toxicity, because we did not find a link between PNPLA3 variants and fibrosis in our patients without HCV co-infection. Since HIV control by cART for longer than one year was an initial inclusion criteria for entering our study, HIV infection and cART constituted the major risk factors for liver disease, while patients with potentially confounding factors such as hepatitis co-infection and obesity were infrequent. Thus, our findings in HIV-positive patients on cART may be a hint that important steps in the pathogenesis of fatty liver disease differ between drug toxicity due to antiretroviral drugs and metabolic stress associated with co-infection, alcoholic and non-alcoholic steatosis.

The *PNPLA3* gene encodes for a triglyceride lipase [27]. Importantly, the physiological role of *PNPLA3* as well as the functional disturbance associated with carriage of the *PNPLA3* 148 M variant are still incompletely understood (see overview in Anstee et al. [15]). It is accepted that the disease-modifying effect of *PNPLA3* is not due to altered insulin sensitivity [28–30]. Likewise, little is known on the precise protein structure and functional role of *TM6SF2*, which by analogy has been proposed to act as a lipid transporter mainly localized in the endoplasmatic reticulum and ER-Golgi intermediate compartments [31]. Knock-down of *TM6SF2*, presumably mimicking the effect of the *TM6SF2* mutation, reduced secretion of triglyceride-rich lipoproteins and resulted in marked cellular lipid accumulation, whereas overexpression of TM6SF2 reduced number and size of intracellular lipid droplets. Of note, *TM6SF2* and *NCAN* are in close genetic linkage. Accordingly, we observed a high concordance between the minor allele frequencies of the two respective SNP’s.

On the other hand, fatty liver disease in HIV infection, initially attributed to a protease-inhibitor-induced lipodystrophy syndrome, is nowadays attributed to the effects of cART in general, recovery of health and accelerated aging. Although insights in this area increase steadily, details of the involved processes are still incomplete [32]. HIV itself and antiviral treatment may contribute to hepatic fat accumulation. However, hepatic steatosis was not associated with prolonged treatment duration.

Of note, our patients with hepatic steatosis were more than three years older than patients without fatty liver disease. The age difference was not statistically significant but could be evidence that accelerated aging of cART-treated patients and age related complications such as diabetes, obesity and reduced insulin resistance, may further contribute to hepatic steatosis in these patients. In addition, HIV itself provokes mitochondrial alterations leading to fatty liver disease and lipodystrophy [25]. Finally, we cannot exclude that other genetic variants, which had not been identified when we performed our study, eventually contribute to the risk of fatty liver disease in HIV infection, analogous to the recent addition of the *MBOAT7* gene to the genetic risk factors in alcohol-related cirrhosis [10]. Better insights into the interactions between HIV infection itself and hepatic metabolic pathways are needed before we can fully understand the differential roles of genetic polymorphisms in fatty liver diseases deriving from different etiologies.

Owing to a rather limited patient number we may have underestimated subtle effects of genetic variants: for example, the presence of a *GCKR* (rs780094) A allele was associated with increased CAP values. Furthermore, the *GCKR* (rs780094) polymorphism correlated with elevated triglycerides, analogous to the findings in other cohorts [33]. Thus, *GCKR* (rs780094) may play some role in the development of steatosis under cART, since this polymorphism seems to influence serum lipid traits in HIV-infected patients.

NAFLD is strongly associated with the metabolic syndrome including insulin resistance, obesity and type 2 diabetes mellitus [34]. Our results are in line with this concept and revealed that HIV positive patients with fatty liver had significantly higher BMI values, suggesting that conventional nutritional factors contribute importantly to fatty liver in HIV-infected patients on cART. However, neither GCKR variants nor any other studied polymorphism was statistically associated with an increased BMI.

There are certain limitations to our study: importantly we did not assess hepatic steatosis, inflammation and fibrosis histologically. It is currently not possible to perform liver biopsy in sufficient numbers of patients with fatty liver disease outside of interventional treatment studies in Germany. Thus, the chosen approach to assess hepatic fatty liver disease by CAP and fibrosis by hepatic stiffness is the second best available method, since both transient elastography and CAP have been validated in large series of studies [35, 36].

In summary, our comprehensive analysis identified conventional metabolic parameters rather than the known genetic variants as risk factors for hepatic steatosis and fibrosis in HIV infected patients on cART. However, genetic polymorphisms may exert negative effects by affecting metabolic parameters such as triglyceride levels, once additional co-factors, e.g. obesity and HCV co-infection, are also present.

## Supporting information

S1 Table Minor allele frequencies. (DOCX)
