# Supplementary material for: Genetic polymorphisms associated with fatty liver disease and fibrosis in HIV positive patients receiving combined antiretroviral therapy (cART)
Source: PLoS One. 2017 Jun 8;12(6):e0178685. doi: 10.1371/journal.pone.0178685 (PMC5464588; doi:10.1371/journal.pone.0178685)
Supplement: S1 Table — (DOCX) [file pone.0178685.s001.docx]

|  |
| --- |
|  |

|  |
| --- |
| \| Allele \| Healthy controls \| HIV pos. with  fatty liver \| HIV pos. with fatty liver \| p-value \| \| --- \| --- \| --- \| --- \| --- \| \| PNPLA 3 \| 22.5 % \| 18.4 % \| 15.4 % \| n.s \| \| NCAN \| 9.1 % \| 5.3 % \| 7.7 % \| n.s \| \| GCKR \| 38.6 % \| 27.6 % \| 38.5 % \| n.s \| \| PPP1R3B \| 8.4 % \| 6.6 % \| 3.8 % \| n.s \| \| LYPLAL \| 15.4 % \| 18.4 % \| 19.2 % \| n.s \| \| TM6SF2 \| 8.7 % \| 5.3 % \| 7.7 % \| n.s \| \| MBOAT \| N.D \| 36.8 % \| 53.8 % \| n.s \| |
